# Genome-based trait prediction in multi- environment breeding trials in groundnut

**DOI:** 10.1007/s00122-020-03658-1

**Published:** 2020-08-18

**Authors:** Manish K. Pandey, Sunil Chaudhari, Diego Jarquin, Pasupuleti Janila, Jose Crossa, Sudam C. Patil, Subramaniam Sundravadana, Dhirendra Khare, Ramesh S. Bhat, Thankappan Radhakrishnan, John M. Hickey, Rajeev K. Varshney

**Affiliations:** 1 International Crops Research Institute for the Semi-Arid Tropics (ICRISAT), Hyderabad, India; 2 International Maize and Wheat Improvement Center (CIMMYT), Mexico City, Mexico; 3 Mahatma Phule Krishi Vidyapeeth (MPKV), Jalgaon, India; 4 Tamil Nadu Agricultural University (TNAU), Coimbatore, India; 5 Jawaharlal Nehru Krishi Vishwa Vidyalaya (JNKVV), Jabalpur, India; 6 University of Agricultural Sciences (UAS)-Dharwad, Dharwad, India; 7 ICAR-Directorate of Groundnut Research (DGR), Junagadh, India; 8 The Roslin Institute, The University of Edinburgh, Edinburgh, Scotland, UK

## Abstract

***Key message*:**

**Comparative assessment identified naïve interaction model, and naïve and informed interaction GS models suitable for achieving higher prediction accuracy in groundnut keeping in mind the high genotype × environment interaction for complex traits.**

**Abstract:**

Genomic selection (GS) can be an efficient and cost-effective breeding approach which captures both small- and large-effect genetic factors and therefore promises to achieve higher genetic gains for complex traits such as yield and oil content in groundnut. A training population was constituted with 340 elite lines followed by genotyping with 58 K ‘Axiom_Arachis’ SNP array and phenotyping for key agronomic traits at three locations in India. Four GS models were tested using three different random cross-validation schemes (CV0, CV1 and CV2). These models are: (1) model 1 (M1 = E + L) which includes the main effects of environment (E) and line (L); (2) model 2 (M2 = E + L + G) which includes the main effects of markers (G) in addition to E and L; (3) model 3 (M3 = E + L + G + GE), a naïve interaction model; and (4) model 4 (E + L + G + LE + GE), a naïve and informed interaction model. Prediction accuracy estimated for four models indicated clear advantage of the inclusion of marker information which was reflected in better prediction accuracy achieved with models M2, M3 and M4 as compared to M1 model. High prediction accuracies (> 0.600) were observed for days to 50% flowering, days to maturity, hundred seed weight, oleic acid, rust@90 days, rust@105 days and late leaf spot@90 days, while medium prediction accuracies (0.400–0.600) were obtained for pods/plant, shelling  %, and total yield/plant. Assessment of comparative prediction accuracy for different GS models to perform selection for untested genotypes, and unobserved and unevaluated environments provided greater insights on potential application of GS breeding in groundnut.

**Electronic supplementary material:**

The online version of this article (10.1007/s00122-020-03658-1) contains supplementary material, which is available to authorized users.

## Introduction

Groundnut (*Arachis hypogaea* L.) is a self-pollinated crop, cultivated in > 100 countries worldwide, and has occupied a global area of 28.5 million ha producing 45.95 million tons with the productivity of 1.61 tons/ha during 2018 (http://www.fao.org/faostat/en/#data/QC). Mostly smallholder farmers are engaged in groundnut cultivation under rainfed conditions with limited resources and inputs in Africa and Asia. Considering the strength of genomics-based robust and precise selection of breeding progenies (Pandey et al. 2012a; Varshney et al. 2013), selection of parents and individuals in the segregating breeding populations can be made more precise and efficient.

Last decade witnessed rapid development of genomic resources such as large scale molecular markers (Wang et al. 2012), genetic maps (Gautami et al. 2012) and genome sequences (Bertioli et al. 2019; Chen et al. 2019; Zhuang et al. 2019) and deployment in genomics-assisted breeding (GAB) in groundnut (see Pandey et al. 2016, 2020; Varshney 2016; Varshney et al. 2019). There are three GAB approaches, namely marker-assisted backcrossing (MABC), marker-assisted recurrent selection (MARS) and genomic selection (GS). MABC and MARS require trait association, while the GS does not need such analysis. Realizing the limitation associated with MABC and MARS to capture small-effect genetic factors, GS has emerged as the most promising, efficient and cost-effective breeding approach which capture both small- and large-effect genetic factors. GS promises to achieve higher genetic gains to improve complex traits (Meuwissen et al. 2001; Heffner et al. 2009; Bernardo 2010; Shikha et al. 2017; Wang et al. 2019) including legumes (Li et al. 2018). GS uses uniformly distributed genetic markers across the genome to predict genomic estimated breeding values (GEBV) using multiple methods with varying degrees of complexity, computational efficiency and predictive accuracy (see Jannink et al. 2010; Desta and Ortiz 2014; Wang et al. 2018). Apart from it, GS is the only modern genomics-based approach with the potential to accumulate thousands of favorable alleles to develop resilient crop lines with high yield potential. This approach has been utilized extensively in livestock breeding (Hays and Goddard 2010; van der Werf 2013; Hays et al. 2013; Meuwissen et al. 2016) and is still evolving in plant breeding. If integrated with rapid generation advancement technology such as speed breeding, the GS can make remarkable achievement and positive impact on breeding programs (Watson et al. 2019) including groundnut (Pandey et al. 2020).

The learnings from genomic prediction strategies from successful animal breeding programs can easily be translated for deployment of genomic prediction-based breeding in crops (Hickey et al. 2017; Xu et al. 2020). In order to fix and evaluate several factors, many studies were conducted to choose appropriate GS models and criteria (Burgueño et al. 2012; Heslot et al. 2012; Jarquín et al. 2014). Such efforts could be seen in last few years in several crop plants such as maize (Sun et al. 2019; Millet et al. 2019), wheat (Song et al. 2017; Norman et al. 2018), rice (Cerrudo et al. 2018; Bhandari et al. 2019), barley (Nielsen et al. 2016), oats (Asoro et al. 2011, 2013), oil palm (Wong and Bernardo 2008) and chickpea (Roorkiwal et al. 2018). In order to enhance precision of predicting GEBVs in the breeding population, it is important to achieve higher correlation between the GEBVs estimated on training population (TP) and in validation sets during cross-validation.

The major problem for the improvement of quantitative traits in crop breeding has been the presence of large genotype × environment interactions (G × E) effects which more often complicate the trait expression by adversely affecting the heritability and response to selection resulting in low genetic gain. G × E effects pose serious challenge to prediction of GEBVs in the GS breeding. Significant variation among different environments is quite obvious due to varied climatic conditions, and it becomes very difficult for optimizing GS models for such environments when complete information across germplasm sets and target environments is not available for use in modeling. In such scenarios, the robust genomic prediction models are required which can take care of G × E interactions to facilitate implementation of GS breeding across germplasm sets and environments. Few GS models were developed by incorporating G × E interaction component either by using structured covariances to model relationships among environments (Burgueño et al. 2012) or by including environmental information to model relationships via covariance structures (Jarquín et al. 2014). Therefore, in order to initiate GS breeding in groundnut, it is utmost important to assess the potential and comparative performance of such promising models by using multi-season phenotyping and high density genotyping data on a sizeable training population. In this context, a training set with 340 diverse and elite groundnut genotypes were extensively phenotyped for important breeding traits and genotyped with high-density ‘Axiom_Arachis’ array containing > 58 K highly informative genome-wide single nucleotide polymorphism (SNP) markers. Four different GS models were tested on this training set with three cross-validation (CV) scenarios mimicking prediction problems such as prediction of tested genotypes in tested environments, untested genotypes in tested environments and tested genotypes in untested environments. The best performing GS models can be used for initiating GS breeding for improving complex traits to achieve higher genetic gains in groundnut.

## Materials and methods

### Constitution of training set and phenotyping

A genomic selection training population (GSTP) was constituted with 340 groundnut genotypes that includes elite breeding lines from the groundnut breeding programs from International Crops Institute for the Semi-Arid Tropics (ICRISAT), Hyderabad; University of Agricultural Sciences (UAS), Dharwad; Indian Council of Agricultural Research-Directorate of Groundnut Research (ICAR-DGR), Junagadh, along with some accessions from gene bank of ICRISAT (that are used in breeding programs) and popular cultivars from India (Supplementary Table 1). This training population includes 227 lines from subspecies *fastigiata* and 113 lines from subspecies *hypogaea* and has variation for key agronomical traits focussed by the Indian groundnut breeding programs. From the perspective of botanical varieties, 212 lines belong to *vulgaris* (Spanish bunch), 111 lines belong to botanical variety *hypogaea*, 10 to *fastigiata* (Valencia), four to *peruviana* and single representative line to *aequatoriana*, *hirsuta* and unknown botanical type (Chaudhari et al. 2019). These lines were phenotyped for 11 agronomic, 7 quality and 6 foliar fungal disease resistance traits at Patancheru, Aliyarnagar and Jalgaon locations in India during two environments (Rainy 2015 and Post-Rainy 2015–2016). The experimental trials were conducted in alpha lattice design with two replications. The detail procedure of conducting trials along with phenotyping of disease resistance at three different time intervals each for rust (rust@75 days, rust@90 days and rust@105 days) and late leaf spot (Late leaf spot@75 days, Late leaf spot@90 days and Late leaf spot@105 days) can be found in Chaudhari et al. (2019). The data on agronomic traits included days to 50% flowering, days to maturity, primary branches/plant, pods/plant, plant height (cm), pod yield/plant (g), shelling  %, hundred seed weight (g), seed yield/plant (g), total yield/plot (g) and pod yield/ha (kg) recorded from both the replications across environments. The oil quality traits including oleic acid, linoleic acid, oleic/linoleic acid ratio, palmitic acid, stearic acid, oil content and protein content were estimated using near-infrared reflectance spectroscopy (NIRS).

### Genotyping with Axiom_Arachis SNP array and SNP allele calling

High-quality genomic DNA was isolated from the plant leaves collected from 15 days old seedlings using high-throughput mini-DNA extraction method (Pandey et al. 2012b). Quality and quantity of DNA were assessed using spectrophotometer (Shimadzu UV160A, Japan). High-density genotyping data have been generated for 318 lines using high-quality DNA samples with Axiom_Arachis SNP array (Pandey et al. 2017; Clevenger et al. 2017) containing 58 K highly informative genome- wide SNPs (Supplementary Table 2). The SNP genotyping on Affymetrix GeneTitan^®^platform and SNP calling has been performed following the methods explained in Pandey et al. (2017). In brief, the target probes were prepared for all the 318 lines followed by amplification, fragmentation, hybridization on the chip, extension through DNA ligation and signal amplification. Staining and scanning the samples were performed on The GeneTitan^®^ Multi- Channel Instrument. The software Axiom™ Analysis Suite version 1.0 was used for allele calling for all the 318 lines of the GSTP. The quality control (QC) analysis of samples was performed using ‘*Best Practices*’ workflow to select samples which passed the QC test. The genotype calls were produced using the ‘*Sample QC*’ workflow followed by using ‘*Genotyping*’ workflow to perform genotyping on the imported CEL files. Finally, the ‘*Summary Only*’ workflow was used to produce a summary and allows to retrieve SNP data for further analysis at the DQC > 0.75 and call rates > 90. The above criteria helped in removing the SNPs with low call rates, thus, keeping only the high-quality SNPs for the further analysis.

### Statistical genomic-enabled prediction models

Total four genomic selection models were tested using the genotyping and phenotyping data on training set as explained in Jarquín et al. (2014) and Roorkiwal et al. (2018). Of these four models, two are main-effect models, and two include genomic × environment interactions. These models are: (1) model 1 (M1 = E + L) which includes the main effects of environments (E) and lines (L); (2) model 2 (M2 = E + L + G) which includes the main effects of markers (G) in addition to environments (E) and lines (L); (3) model 3 (M3 = E + L + G + GE), a naïve interaction model; and (4) model 4 (E + L + G + LE + GE), a naïve and informed interaction model.

The Bayesian Generalized Linear Regression (BGLR) R-package **(**de los Campos et al. 2013; Pérez-Rodríguez et al. 2015) was used for performing entire analysis with these four GS models. The scripts for these four GS models have already been made available in public domain by Pérez-Rodríguez et al. (2015), and technical details for these GS models are provided in Roorkiwal et al. (2018). A brief statistical description of the four models (M1–M4) is given below in addition to the conventional base line model. In the base line model, the response of the *j*th (*j* = 1,…,*J*) genotype evaluated in the *i*th (*i* = 1,…,*I*) environment $\{ y_{ij} \}$ is the sum of an overall mean *μ* plus random deviations around zero due to environmental $E_{i} \sim N(0, \sigma_{E}^{2} )$ that is assumed to have a normal distribution with mean 0 and variance $\sigma_{E}^{2}$ assuming an independent and identically distributed response (IID), and line effects are assumed idd $L_{j} \sim N(0, \sigma_{L}^{2} )$ where $\sigma_{L}^{2}$ is the variance of the lines, and the interaction between the *i*th genotype and the *j*th environment is also iid $LE_{ji} \sim N(0, \sigma_{LE}^{2} )$ where $\sigma_{LE}^{2}$ is the interaction variance and the random error term is assumed iid $e_{ji} \sim N(0, \sigma_{e}^{2} )$

$$ y_{ij} = \mu + E_{i} + L_{j} + EL_{ij} + e_{ij} $$

Evidently, this model does not allow borrowing of information among lines because they were treated as independent outcomes. The following models were derived from the baseline model by either subtracting terms or modifying the underlying assumptions.

#### Model 1 (M1): environment + line main effects (E + L)

This model is obtained from the baseline model by retaining the first three components, while their underlying assumptions remain unchanged.

1 $$ y_{ij} = \mu + E_{i} + L_{j} + e_{ij} $$

#### Model 2 (M2): environment + line + genomic main effects (E + L + G)

Adding to model M1 as a linear combination between markers and their correspondent marker effects, $g_{j} = \sum\nolimits_{m = 1}^{p} {x_{jm} b_{m} }$, genomic information can be introduced using the following linear predictor

2 $$ y_{ij} = \mu + E_{i} + L_{j} + g_{j} + e_{ij} $$

where $b_{m} \mathop \sim\limits^{iid} N(0,\sigma_{b}^{2} )$ represents the random effect of the *m*th (*m* = 1,…,*p*) marker and $\sigma_{b}^{2}$ its correspondent variance component. Using the results from the multivariate normal distribution, ${\mathbf{g}} = ( {\text{g}}_{1} , \ldots ,{\text{g}}_{J} )^{{\prime }}$, the vector of genetic effects, follows a normal density with zero mean vector and covariance matrix ${\text{Cov}}({\mathbf{g}}) = {\mathbf{G}}\sigma_{g}^{2}$ with ${\mathbf{G}} = \frac{{{\text{XX}}^{{\prime }} }}{p}$ as the genomic relationship matrix. It describes genetic similarities among pairs of individuals. Here, X represents the centered and standardized (by columns) genomic matrix and $\sigma_{g}^{2} = p \times \sigma_{b}^{2}$ acts as the correspondent variance component such that ${\mathbf{g}} = \{ {\text{g}}_{j} \} \sim N(0,{\mathbf{G}}\sigma_{{\mathbf{g}}}^{2} )$. In this model, the line effect *L*_*j*_ is retained in the model to account for imperfect information and model mis-specification due to imperfect linkage disequilibrium.

#### Model 3 (M3): environment + line + genomic + genomic × environment interaction [E + L + G + (G × E)]

This model extends model M3 by adding the genomic × environment interaction as follows:

3 $$ y_{ij} = \mu + E_{i} + L_{j} + g_{j} + Eg_{ij} + e_{ij} $$

The main disadvantage of the previous models is that they only consider the main effect of the lines/genotypes across environments, avoiding specific responses of each genotype in each environment. To overcome this issue, the G × E interaction is introduced via covariance structures, as shown by Jarquín et al. (2014). Here, interaction component $EL_{ij}$ is replaced by $Eg_{ij}$, where $\varvec{Eg} = \{ Eg_{ij} \} \sim N(0,(\varvec{Z}_{\varvec{g}} \varvec{GZ}_{\varvec{g}}^{{\prime }} )^\circ (\varvec{Z}_{\varvec{E}} \varvec{Z}_{\varvec{E}}^{{\prime }} )\sigma_{{\varvec{Eg}}}^{2} )$ and $\varvec{Z}_{\varvec{g}}$ and $\varvec{Z}_{\varvec{E}}$ are the correspondent incidence matrices for genotypes and environments, $\sigma_{Eg}^{2}$ is the associated variance component for this interaction, and ‘$\circ$’ represents the Hadamard or Schur product (element-to-element product) between two matrices.

#### Model 4 (M4): environment + line + genomic + genomic × environment + line × environment interaction [E + L + G + (G × E) + L × E)]

This model extends model M2 by adding the line × environment interaction as follows:

$$ y_{ij} = \mu + L_{J} + E_{i} + LE_{ij} + g_{j} + Eg_{ij} + e_{ij} $$

where all the terms have been previously defined.

### Assessing different prediction problems using various cross-validation strategies

The above-mentioned four GS models (E + L, E + L + G, E + L + G + GE and E + L + G + LE + GE) were deployed in training set using three different random cross-validation (CV) schemes, namely CV0, CV1 and CV2. Random CV2 represents incomplete field trials where some lines are observed in some environments but not in others; the goal here is to predict the crop performance of these lines in environments where these lines have not yet been phenotyped. Random CV1 predicts newly developed lines to measure the predictive ability of new lines that have not yet been phenotyped in any field, predictive ability between observed and unobserved genotypes is based on genetic similarities as main source of information, and CV0 is the prediction of already observed lines in unobserved environments (CV0). In CV0, the main interest is to predict the crop performance of lines in potentially new environments.

For random cross-validation CV1 and CV2, the prediction accuracies of the four models were computed by performing random fivefold cross-validation where the performance of 20% of the lines (testing set) was predicted considering the remaining 80% observed lines as training set. For CV1, none of the 20% of the lines in the testing set were observed in any of the environments (combination), whereas for CV2, the 20% of the lines in the testing set were observed in some environments but not in the others. The prediction accuracy is obtained as the average Pearson’s correlations between the observed breeding values and predicted GEBVs.

## Results

### Identification of genetic polymorphism and phenotypic variation in training population

Genotyping data with SNP array have been generated on 318 lines, while phenotyping data were generated for 340 lines. Genotyping on 318 lines with Axiom_Arachis SNP array identified 13,355 polymorphic SNPs. The phenotypic data generated on 340 lines showed wide genetic variation for different agronomical, quality and foliar disease resistance traits. All the 11 agronomic traits have shown high (75–90%) to very high (> 90%) heritability, namely days to maturity (96.6%), hundred seed weight (93.4%), plant height (92.3%), yield/ha (89.7%), total yield/plant (89.3%), pod yield/plant (85.8), pods/plant (85.0%), and days to 50% flowering (84.8%), seed yield/plant (84.6%), shelling  % (82.9%) and primary branches/plant (78.7%) (Supplementary Table 3). In case of 7 quality traits, the highest heritability was observed for oleic/linoleic acid ratio (96.7%) followed by palmitic acids (84.0%), oleic acid (82.1%), linoleic acid (81.7%), oil content (78.6%), stearic acid (77.5%) and protein content (57.4%) recorded medium heritability. The foliar disease resistance traits recorded high heritability at different days of sowing (80.4% for rust@75 days, 84.2% for rust@90 days, 82.7% for rust@105 days, 83.9% for LLS@90 days, 79.7% for LLS@105 days and 74.5% for LLS@75 days).

### Comparative performance of four GS models under three cross-validation schemes

**Fig. 1:**
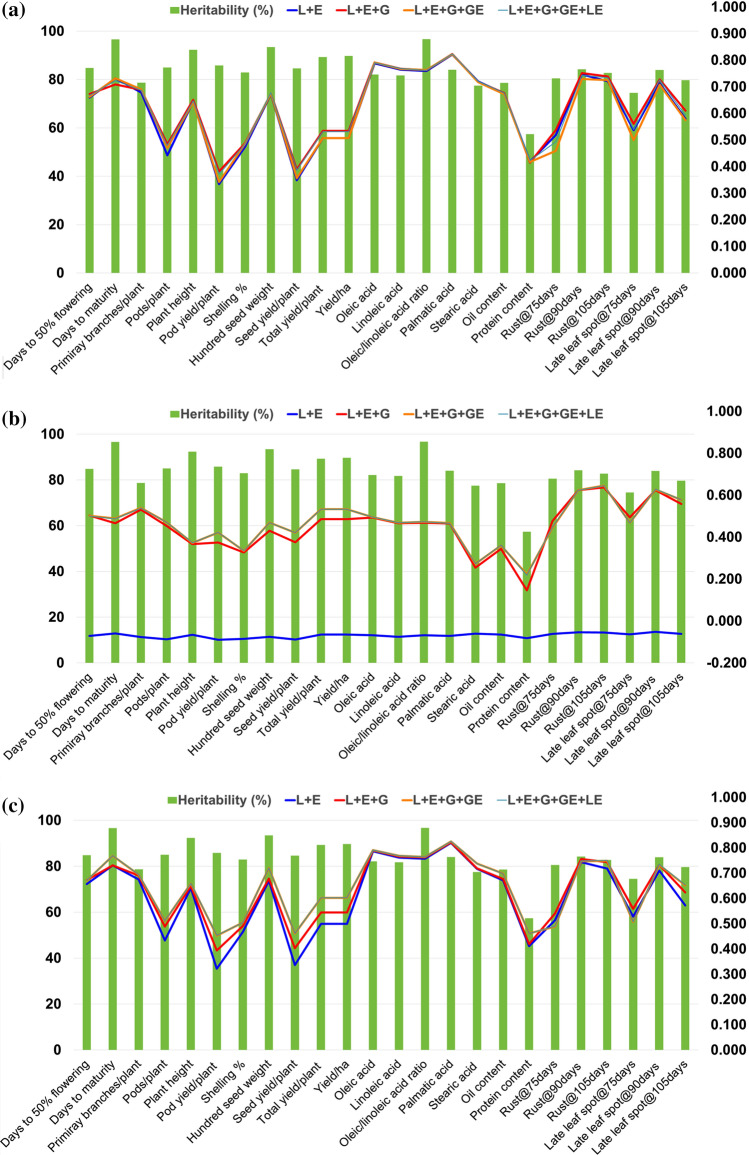
Cross-validation between the predicted and the observed values for **a** unobserved environment (CV0); **b** untested genotypes (CV1); and unevaluated environment (CV2) for different agronomic, quality and disease resistance traits of groundnut

**Fig. 2:**
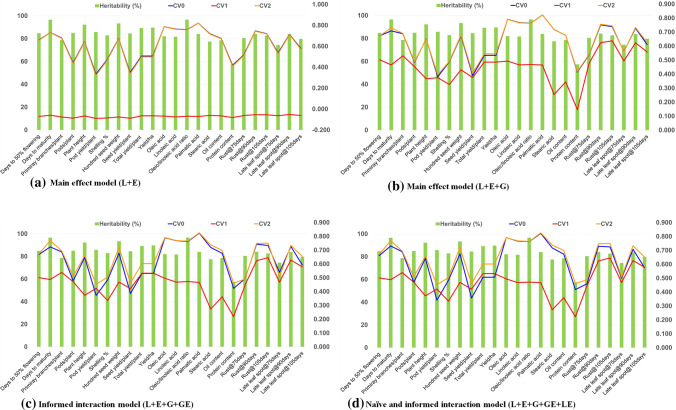
Comparative performance of four genomic prediction models in three different cross-validation scenarios in groundnut training population

Prediction accuracy estimated by four models indicated clear advantage of the inclusion of marker information which was reflected in better prediction accuracy achieved from models E + L + G, informed interaction (E + L + G + GE) and naïve and informed interaction as compared to E + L model. The detailed results for scheme CV0 (Table 1; Fig. 1a), CV1 (Table 2; Fig. 1b) and CV2 (Table 3; Fig. 1c) are summarized in Table 4 and Fig. 2.

**Table 1:** Mean correlations from tenfold cross-validation between the predicted and the observed values for four models (M1–M4) for unobserved environment (CV0) in different agronomic, quality and disease resistance traits of groundnut

Traits	Main-effect models										Informed interaction model					Naïve and informed interaction model				
	Lines and environment (E + L)					Lines, environment and marker information (E + L + G)					Interaction effects (E + L + G + GE)					Interaction effects (E + L + G + GE + LE)				
	Env1	Env2	Env3	Env4	Mean	Env1	Env2	Env3	Env4	Mean	Env1	Env2	Env3	Env4	Mean	Env1	Env2	Env3	Env4	Mean
Days to 50% flowering (FLOW50)	0.782	0.430	0.722	0.703	0.659	0.801	0.430	0.749	0.713	0.673	0.786	0.429	0.722	0.709	0.662	0.791	0.432	0.735	0.714	0.667
Days to maturity (DM)	0.808	0.886	0.880	0.355	0.732	0.787	0.850	0.833	0.365	0.709	0.807	0.888	0.879	0.352	0.731	0.798	0.875	0.867	0.352	0.723
Primary branches/plant (NPB)	0.683	0.681	0.756	0.594	0.678	0.711	0.676	0.756	0.611	0.688	0.706	0.680	0.766	0.608	0.690	0.709	0.681	0.752	0.605	0.687
Pods/plant (NPP)	0.376	0.492	0.538	0.363	0.442	0.394	0.542	0.599	0.402	0.484	0.395	0.535	0.553	0.397	0.470	0.388	0.536	0.593	0.400	0.479
Plant height (PH)	0.753	0.549	0.733	0.546	0.645	0.765	0.540	0.760	0.538	0.651	0.757	0.544	0.725	0.544	0.643	0.761	0.547	0.739	0.540	0.647
Pod yield/plant (PYPP)	0.267	0.391	0.452	0.227	0.334	0.295	0.426	0.570	0.234	0.381	0.285	0.416	0.419	0.245	0.341	0.285	0.415	0.545	0.243	0.372
Shelling % (SHP)	0.475	0.561	0.437	0.425	0.474	0.461	0.607	0.420	0.464	0.488	0.471	0.593	0.430	0.447	0.485	0.471	0.595	0.425	0.448	0.485
Hundred seed weight (HSW)	0.712	0.744	0.729	0.509	0.673	0.724	0.739	0.731	0.504	0.674	0.720	0.747	0.726	0.508	0.675	0.726	0.740	0.740	0.507	0.678
Seed yield/plant (SYPP)	0.285	0.391	0.455	0.262	0.348	0.298	0.436	0.555	0.269	0.389	0.304	0.418	0.423	0.280	0.356	0.305	0.419	0.540	0.281	0.386
Total yield/plant (TYPLT)	0.507	0.521	0.674	0.326	0.506	0.537	0.572	0.717	0.311	0.534	0.530	0.549	0.619	0.331	0.507	0.531	0.558	0.699	0.337	0.531
Yield/ha (YPH)	0.507	0.521	0.674	0.326	0.506	0.537	0.572	0.717	0.311	0.534	0.530	0.549	0.619	0.331	0.507	0.531	0.558	0.699	0.337	0.531
Oleic acid (OA)	0.788	0.807	0.860	0.698	0.788	0.791	0.813	0.862	0.699	0.791	0.791	0.813	0.864	0.699	0.792	0.790	0.811	0.861	0.697	0.790
Linoleic acid (LA)	0.768	0.785	0.839	0.664	0.763	0.767	0.795	0.842	0.665	0.767	0.768	0.793	0.841	0.666	0.767	0.768	0.795	0.843	0.667	0.769
Oleic/linoleic acid ratio (OLR)	0.771	0.786	0.836	0.643	0.759	0.774	0.792	0.839	0.647	0.763	0.774	0.792	0.840	0.646	0.763	0.774	0.790	0.837	0.645	0.762
Palmitic acid (PA)	0.832	0.813	0.862	0.777	0.821	0.834	0.815	0.865	0.777	0.823	0.834	0.814	0.864	0.777	0.822	0.834	0.816	0.865	0.779	0.823
Stearic acid (SA)	0.730	0.764	0.765	0.620	0.719	0.721	0.761	0.775	0.618	0.719	0.731	0.763	0.758	0.617	0.717	0.730	0.764	0.765	0.621	0.720
Oil content (OC)	0.698	0.730	0.756	0.514	0.674	0.694	0.732	0.774	0.505	0.676	0.697	0.728	0.752	0.512	0.672	0.695	0.735	0.764	0.514	0.677
Protein content (PC)	0.465	0.481	0.457	0.273	0.418	0.460	0.490	0.438	0.271	0.415	0.465	0.485	0.447	0.274	0.418	0.470	0.478	0.460	0.284	0.423
Rust@75 days (RUST75)	0.741	0.086	0.731	NA	0.518	0.784	0.099	0.730	NA	0.538	0.678	0.099	0.601	NA	0.459	0.680	0.098	0.703	NA	0.494
Rust@90 days (RUST90)	0.819	0.593	0.827	NA	0.746	0.844	0.592	0.818	NA	0.752	0.802	0.591	0.796	NA	0.730	0.812	0.595	0.823	NA	0.744
Rust@105 days (RUST105)	0.709	0.656	0.796	NA	0.720	0.731	0.669	0.817	NA	0.739	0.712	0.663	0.799	NA	0.725	0.720	0.670	0.812	NA	0.734
Late leaf spot@75 days (LLS75)	0.704	0.189	0.718	NA	0.537	0.726	0.209	0.748	NA	0.561	0.648	0.188	0.661	NA	0.499	0.703	0.199	0.716	NA	0.539
Late leaf spot@90 days (LLS90)	0.770	0.583	0.787	NA	0.713	0.787	0.589	0.807	NA	0.728	0.752	0.579	0.793	NA	0.708	0.781	0.592	0.805	NA	0.726
Late leaf spot@105 days (LLS105)	0.566	0.497	0.675	NA	0.579	0.599	0.514	0.717	NA	0.610	0.550	0.504	0.670	NA	0.575	0.572	0.516	0.691	NA	0.593

Environment1 (ENV1): Aliyarnagar_Rainy 2015; Environment2 (ENV2): Jalgoan_Rainy 2015; Environment3 (ENV3): ICRISAT_Rainy 2015; Environment4 (ENV4):ICRISAT Post-Rainy 2015

**Table 2:** Mean correlations from tenfold cross-validation between the predicted and the observed values for four models (M1–M4) for untested some lines (CV1) in different agronomic, quality and disease resistance traits of groundnut

Traits	Main-effect models										Informed interaction model					Naïve and informed interaction model				
	Lines and environment (E + L)					Lines, environment and marker information (E + L + G)					Interaction effects (E + L + G + GE)					Interaction effects (E + L + G + GE + LE)				
	Env1	Env2	Env3	Env4	Mean	Env1	Env2	Env3	Env4	Mean	Env1	Env2	Env3	Env4	Mean	Env1	Env2	Env3	Env4	Mean
Days to 50% flowering (FLOW50)	− 0.062	− 0.072	− 0.086	− 0.064	− 0.071	0.606	0.287	0.585	0.534	0.503	0.616	0.242	0.601	0.549	0.502	0.614	0.244	0.598	0.547	0.501
Days to maturity (DM)	− 0.056	− 0.064	− 0.063	− 0.052	− 0.059	0.414	0.459	0.434	0.557	0.466	0.437	0.466	0.442	0.613	0.489	0.434	0.464	0.440	0.612	0.488
Primary branches/plant (NPB)	− 0.052	− 0.089	− 0.072	− 0.092	− 0.077	0.589	0.479	0.554	0.501	0.531	0.608	0.475	0.562	0.514	0.540	0.605	0.474	0.561	0.513	0.538
Pods/plant (NPP)	− 0.106	− 0.091	− 0.092	− 0.059	− 0.087	0.356	0.497	0.545	0.414	0.453	0.367	0.514	0.538	0.464	0.471	0.369	0.513	0.539	0.465	0.471
Plant height (PH)	− 0.067	− 0.077	− 0.066	− 0.053	− 0.066	0.457	0.236	0.503	0.273	0.367	0.488	0.213	0.515	0.275	0.373	0.485	0.217	0.513	0.277	0.373
Pod yield/plant (PYPP)	− 0.098	− 0.118	− 0.090	− 0.052	− 0.089	0.278	0.406	0.583	0.231	0.374	0.283	0.475	0.590	0.341	0.422	0.286	0.474	0.591	0.341	0.423
Shelling % (SHP)	− 0.078	− 0.097	− 0.103	− 0.064	− 0.085	0.267	0.445	0.213	0.380	0.326	0.287	0.447	0.231	0.371	0.334	0.289	0.448	0.231	0.373	0.335
Hundred seed weight (HSW)	− 0.065	− 0.079	− 0.066	− 0.092	− 0.076	0.526	0.454	0.492	0.250	0.430	0.564	0.486	0.533	0.295	0.469	0.563	0.485	0.532	0.295	0.469
Seed yield/plant (SYPP)	− 0.096	− 0.116	− 0.085	− 0.056	− 0.088	0.255	0.416	0.566	0.264	0.375	0.260	0.470	0.578	0.375	0.421	0.263	0.470	0.578	0.375	0.422
Total yield/plant (TYPLT)	− 0.052	− 0.081	− 0.070	− 0.056	− 0.065	0.482	0.550	0.636	0.276	0.486	0.496	0.598	0.651	0.386	0.533	0.496	0.598	0.651	0.387	0.533
Yield/ha (YPH)	− 0.052	− 0.081	− 0.070	− 0.056	− 0.065	0.482	0.550	0.636	0.276	0.486	0.496	0.598	0.651	0.386	0.533	0.496	0.598	0.651	0.387	0.533
Oleic acid (OA)	− 0.070	− 0.075	− 0.069	− 0.057	− 0.068	0.486	0.520	0.549	0.416	0.493	0.492	0.516	0.551	0.422	0.495	0.493	0.518	0.552	0.423	0.496
Linoleic acid (LA)	− 0.091	− 0.075	− 0.065	− 0.068	− 0.075	0.451	0.517	0.520	0.376	0.466	0.456	0.511	0.518	0.387	0.468	0.456	0.511	0.517	0.389	0.468
Oleic/linoleic acid ratio (OLR)	− 0.073	− 0.061	− 0.072	− 0.067	− 0.068	0.460	0.499	0.525	0.390	0.469	0.466	0.498	0.524	0.401	0.472	0.466	0.499	0.525	0.401	0.473
Palmitic acid (PA)	− 0.085	− 0.081	− 0.061	− 0.056	− 0.071	0.457	0.466	0.509	0.429	0.465	0.465	0.457	0.515	0.433	0.468	0.462	0.457	0.513	0.431	0.466
Stearic acid (SA)	− 0.063	− 0.041	− 0.067	− 0.072	− 0.061	0.170	0.245	0.410	0.193	0.254	0.178	0.233	0.441	0.237	0.272	0.180	0.233	0.438	0.240	0.273
Oil content (OC)	− 0.077	− 0.059	− 0.061	− 0.060	− 0.065	0.329	0.401	0.483	0.163	0.344	0.354	0.424	0.507	0.156	0.360	0.355	0.424	0.507	0.163	0.362
Protein content (PC)	− 0.083	− 0.082	− 0.085	− 0.077	− 0.081	0.124	0.228	0.150	0.083	0.146	0.149	0.283	0.303	0.152	0.222	0.143	0.280	0.297	0.154	0.219
Rust@75 days (RUST75)	− 0.074	− 0.024	− 0.086	NA	− 0.061	0.691	0.113	0.627	NA	0.477	0.692	0.031	0.612	NA	0.445	0.692	0.039	0.612	NA	0.448
Rust@90 days (RUST90)	− 0.069	− 0.040	− 0.054	NA	− 0.054	0.715	0.470	0.688	NA	0.624	0.714	0.462	0.695	NA	0.624	0.714	0.461	0.695	NA	0.623
Rust@105 days (RUST105)	− 0.063	− 0.056	− 0.044	NA	− 0.055	0.627	0.591	0.695	NA	0.638	0.621	0.613	0.702	NA	0.646	0.622	0.613	0.701	NA	0.645
Late leaf spot@75 days (LLS75)	− 0.067	− 0.037	− 0.088	NA	− 0.064	0.631	0.204	0.648	NA	0.494	0.632	0.131	0.642	NA	0.468	0.632	0.128	0.643	NA	0.468
Late leaf spot@90 days (LLS90)	− 0.057	− 0.054	− 0.045	NA	− 0.052	0.664	0.505	0.702	NA	0.624	0.657	0.506	0.718	NA	0.627	0.659	0.510	0.717	NA	0.629
Late leaf spot@105 days (LLS105)	− 0.053	− 0.081	− 0.052	NA	− 0.062	0.534	0.509	0.630	NA	0.558	0.530	0.556	0.647	NA	0.578	0.533	0.557	0.646	NA	0.579

Environment1 (ENV1):Aliyarnagar_Rainy 2015; Environment2 (ENV2):Jalgoan_Rainy 2015; Environment3 (ENV3):ICRISAT_Rainy 2015; Environment4 (ENV4):ICRISAT Post-Rainy 2015

**Table 3:** Mean correlations from tenfold cross-validation between the predicted and the observed values for four models (M1–M4) for some lines evaluated in some environments (CV2) in different agronomic, quality and disease resistance traits of groundnut

Traits	Main-effect models										Informed interaction model					Naïve and informed interaction model				
	Lines and environment (E + L)					Lines, environment and marker information (E + L + G)					Interaction effects (E + L + G + GE)					Interaction effects (E + L + G + GE + LE)				
	Env1	Env2	Env3	Env4	Mean	Env1	Env2	Env3	Env4	Mean	Env1	Env2	Env3	Env4	Mean	Env1	Env2	Env3	Env4	Mean
Days to 50% flowering (FLOW50)	0.780	0.428	0.720	0.699	0.657	0.800	0.426	0.747	0.715	0.672	0.798	0.408	0.756	0.720	0.671	0.800	0.410	0.756	0.719	0.671
Days to maturity (DM)	0.807	0.885	0.880	0.351	0.731	0.794	0.864	0.854	0.411	0.731	0.824	0.858	0.857	0.538	0.769	0.825	0.856	0.855	0.539	0.769
Primary branches/plant (NPB)	0.680	0.675	0.754	0.589	0.675	0.714	0.674	0.760	0.614	0.690	0.719	0.669	0.760	0.628	0.694	0.720	0.669	0.761	0.628	0.695
Pods/plant (NPP)	0.367	0.482	0.532	0.355	0.434	0.398	0.550	0.597	0.408	0.488	0.425	0.571	0.602	0.445	0.511	0.426	0.570	0.602	0.446	0.511
Plant height (PH)	0.750	0.541	0.729	0.541	0.640	0.767	0.533	0.757	0.538	0.649	0.785	0.530	0.763	0.557	0.659	0.785	0.530	0.764	0.558	0.659
Pod yield/plant (PYPP)	0.254	0.374	0.442	0.216	0.321	0.300	0.450	0.583	0.244	0.394	0.323	0.527	0.628	0.331	0.452	0.326	0.527	0.628	0.334	0.454
Shelling % (SHP)	0.470	0.553	0.431	0.418	0.468	0.470	0.601	0.424	0.468	0.491	0.498	0.605	0.445	0.470	0.505	0.496	0.606	0.445	0.471	0.504
Hundred seed weight (HSW)	0.708	0.740	0.727	0.504	0.670	0.731	0.742	0.739	0.504	0.679	0.778	0.769	0.786	0.551	0.721	0.778	0.769	0.786	0.550	0.721
Seed yield/plant (SYPP)	0.274	0.375	0.444	0.253	0.336	0.303	0.456	0.570	0.284	0.403	0.325	0.522	0.617	0.380	0.461	0.328	0.522	0.618	0.382	0.462
Total yield/plant (TYPLT)	0.501	0.511	0.669	0.316	0.499	0.546	0.586	0.723	0.323	0.545	0.575	0.644	0.750	0.438	0.602	0.577	0.644	0.750	0.439	0.603
Yield/ha (YPH)	0.501	0.511	0.669	0.316	0.499	0.546	0.586	0.723	0.323	0.545	0.575	0.644	0.750	0.438	0.602	0.577	0.644	0.750	0.439	0.603
Oleic acid (OA)	0.787	0.806	0.859	0.695	0.787	0.789	0.812	0.863	0.698	0.791	0.790	0.807	0.865	0.703	0.791	0.790	0.808	0.865	0.704	0.792
Linoleic acid (LA)	0.765	0.783	0.838	0.662	0.762	0.766	0.794	0.842	0.663	0.766	0.769	0.792	0.841	0.674	0.769	0.769	0.793	0.840	0.675	0.769
Oleic/linoleic acid ratio (OLR)	0.770	0.785	0.835	0.639	0.757	0.772	0.791	0.840	0.647	0.762	0.773	0.789	0.838	0.657	0.764	0.773	0.790	0.838	0.658	0.765
Palmitic acid (PA)	0.831	0.812	0.861	0.776	0.820	0.834	0.814	0.865	0.776	0.822	0.836	0.812	0.877	0.777	0.825	0.836	0.813	0.876	0.777	0.826
Stearic acid (SA)	0.728	0.761	0.761	0.618	0.717	0.722	0.760	0.778	0.617	0.719	0.741	0.756	0.810	0.645	0.738	0.741	0.756	0.810	0.644	0.738
Oil content (OC)	0.694	0.727	0.753	0.512	0.672	0.694	0.736	0.774	0.501	0.676	0.709	0.762	0.798	0.525	0.699	0.709	0.762	0.798	0.526	0.698
Protein content (PC)	0.458	0.471	0.450	0.264	0.411	0.461	0.493	0.453	0.269	0.419	0.462	0.529	0.537	0.317	0.461	0.462	0.527	0.536	0.317	0.461
Rust@75 days (RUST75)	0.735	0.084	0.721	NA	0.513	0.788	0.106	0.727	NA	0.541	0.767	0.010	0.690	NA	0.489	0.766	0.017	0.689	NA	0.491
Rust@90 days (RUST90)	0.816	0.591	0.824	NA	0.744	0.845	0.590	0.835	NA	0.756	0.830	0.578	0.833	NA	0.747	0.830	0.583	0.832	NA	0.748
Rust@105 days (RUST105)	0.707	0.653	0.793	NA	0.718	0.732	0.678	0.822	NA	0.744	0.725	0.704	0.819	NA	0.749	0.727	0.706	0.818	NA	0.751
Late leaf spot@75 days (LLS75)	0.699	0.186	0.699	NA	0.528	0.732	0.208	0.735	NA	0.559	0.735	0.091	0.694	NA	0.507	0.733	0.096	0.693	NA	0.508
Late leaf spot@90 days (LLS90)	0.767	0.579	0.784	NA	0.710	0.786	0.592	0.820	NA	0.733	0.778	0.589	0.832	NA	0.733	0.779	0.596	0.830	NA	0.735
Late leaf spot@105 days (LLS105)	0.558	0.489	0.670	NA	0.572	0.601	0.542	0.727	NA	0.623	0.599	0.614	0.742	NA	0.652	0.604	0.616	0.742	NA	0.654

Environment1 (ENV1): Aliyarnagar_Rainy 2015; Environment2 (ENV2): Jalgoan_Rainy 2015; Environment3 (ENV3): ICRISAT_Rainy 2015; Environment4 (ENV4): ICRISAT Post- Rainy 2015

**Table 4:** Comparative prediction accuracy by four models (M1 = E + L, M2 = E + L + G, M3 = E + L + G + GE and M4 = E + L + G + GE + LE) and three cross-validation schemes (CV0, CV1 and CV2) in groundnut

Traits	Heritability (%)	Main-effect models								Informed interaction model				Naïve and informed interaction model			
		Lines and environment (E + L)				Lines, environment and marker information E + L + G)				Interaction effects (E + L + G + GE)				Interaction effects (E + L + G + GE + LE)			
		CV0	CV1	CV2	Mean	CV0	CV1	CV2	Mean	CV0	CV1	CV2	Mean	CV0	CV1	CV2	Mean
Days to 50% flowering (FLOW50)	84.8	0.659	− 0.071	0.657	0.415	0.673	0.503	0.672	0.616	0.662	0.502	0.671	0.611	0.668	0.501	0.671	0.613
Days to maturity (DM)	96.6	0.732	− 0.059	0.731	0.468	0.709	0.466	0.731	0.635	0.731	0.489	0.769	0.663	0.723	0.488	0.769	0.660
Primary branches/plant (NPB)	78.7	0.679	− 0.077	0.675	0.426	0.688	0.531	0.690	0.637	0.690	0.540	0.694	0.641	0.687	0.538	0.695	0.640
Pods/plant (NPP)	85.0	0.442	− 0.087	0.434	0.263	0.484	0.453	0.488	0.475	0.470	0.471	0.511	0.484	0.479	0.471	0.511	0.487
Plant height (PH)	92.3	0.645	− 0.066	0.640	0.407	0.651	0.367	0.649	0.556	0.643	0.373	0.659	0.558	0.647	0.373	0.659	0.560
Pod yield/plant (PYPP)	85.8	0.334	− 0.089	0.321	0.189	0.381	0.374	0.394	0.383	0.341	0.422	0.452	0.405	0.372	0.423	0.454	0.416
Shelling % (SHP)	82.9	0.475	− 0.085	0.468	0.286	0.488	0.326	0.491	0.435	0.485	0.334	0.505	0.441	0.485	0.335	0.504	0.441
Hundred seed weight (HSW)	93.4	0.673	− 0.076	0.670	0.423	0.674	0.430	0.679	0.595	0.675	0.469	0.721	0.622	0.678	0.469	0.721	0.623
Seed yield/plant (SYPP)	84.6	0.348	− 0.088	0.336	0.199	0.389	0.375	0.403	0.389	0.356	0.421	0.461	0.413	0.386	0.422	0.462	0.423
Total yield/plant (TYPLT)	89.3	0.507	− 0.065	0.499	0.314	0.534	0.486	0.545	0.521	0.507	0.533	0.602	0.547	0.531	0.533	0.603	0.556
Yield/ha (YPH)	89.7	0.507	− 0.065	0.499	0.314	0.534	0.486	0.545	0.521	0.507	0.533	0.602	0.547	0.531	0.533	0.603	0.556
Oleic acid (OA)	82.1	0.788	− 0.068	0.787	0.502	0.791	0.493	0.791	0.692	0.792	0.495	0.791	0.693	0.790	0.496	0.792	0.693
Linoleic acid (LA)	81.7	0.764	− 0.075	0.762	0.484	0.767	0.466	0.766	0.666	0.767	0.468	0.769	0.668	0.769	0.468	0.769	0.669
Oleic/linoleic acid ratio (OLR)	96.7	0.759	− 0.068	0.757	0.483	0.763	0.469	0.762	0.665	0.763	0.472	0.764	0.666	0.762	0.473	0.765	0.666
Palmitic acid (PA)	84.0	0.821	− 0.071	0.820	0.524	0.823	0.465	0.822	0.703	0.822	0.468	0.825	0.705	0.823	0.466	0.826	0.705
Stearic acid (SA)	77.5	0.720	− 0.061	0.717	0.459	0.719	0.254	0.719	0.564	0.717	0.272	0.738	0.576	0.720	0.273	0.738	0.577
Oil content (OC)	78.6	0.675	− 0.065	0.672	0.427	0.676	0.344	0.676	0.566	0.672	0.360	0.699	0.577	0.677	0.362	0.698	0.579
Protein content (PC)	57.4	0.419	− 0.081	0.411	0.249	0.415	0.146	0.419	0.327	0.418	0.222	0.461	0.367	0.423	0.219	0.461	0.367
Rust@75 days (RUST75)	80.5	0.519	− 0.061	0.513	0.324	0.538	0.477	0.541	0.518	0.459	0.445	0.489	0.464	0.494	0.448	0.491	0.477
Rust@90 days (RUST90)	84.2	0.747	− 0.054	0.744	0.479	0.752	0.624	0.756	0.711	0.730	0.624	0.747	0.700	0.744	0.623	0.748	0.705
Rust@105 days (RUST105)	82.7	0.721	− 0.055	0.718	0.461	0.739	0.638	0.744	0.707	0.725	0.646	0.749	0.707	0.734	0.645	0.751	0.710
Late leaf spot@75 days (LLS75)	74.5	0.537	− 0.064	0.528	0.334	0.561	0.494	0.559	0.538	0.499	0.468	0.507	0.491	0.539	0.468	0.508	0.505
Late leaf spot@90 days (LLS90)	83.9	0.713	− 0.052	0.710	0.457	0.728	0.624	0.733	0.695	0.708	0.627	0.733	0.689	0.726	0.629	0.735	0.697
Late leaf spot@105 days (LLS105)	79.7	0.579	− 0.062	0.572	0.363	0.610	0.558	0.623	0.597	0.575	0.578	0.652	0.601	0.593	0.579	0.654	0.608

#### Performance of four GS models for unobserved environment (CV0 scheme)

In general, the prediction values across four environments with four GS models were found consistent for CV0 scheme (Table 1). The exceptions in consistent prediction with all the four models were observed for days to 50% flowering for Env2 (Jalgaon, Rainy 2015), and days to maturity, hundred seed weight, total yield/plant, yield/ha, oil content, protein content for Env4 (Patancheru, Post-Rainy 2015–2016) (Table 1).

The high prediction accuracy (> 0.600) across the four models was observed for days to 50% flowering (0.659–0.673), days to maturity (0.709–0.732), primary branches/plant (0.679–0.690), plant height (0.643–0.647), hundred seed weight (0.673–0.678), oleic acid (0.788–0.792), linoleic acid (0.764–0.769), OLR (0.759–0.763), palmitic acid (0.821–0.823), stearic acid (0.717–0.720), oil content (0.672–0.677), rust@90 days (0.730–0.752), rust@105 days (0.721–0.739) and late leaf spot@90 days (0.708–0.728) (Tables 1, 4). The traits, namely pods/plant (0.442–0.484), shelling  % (0.475–0.485), total yield/plant (0.507–0.534), yield/ha (0.507–0.534), protein content (0.415–0.423), rust@75 days (0.459–0.538), late leaf spot@75 days (0.499–0.538) and late leaf spot@105 days (0.507–0.534), have obtained medium (0.400–0.600) prediction accuracy. The two important traits in breeding program, pod yield/plant (0.334–0.381) and seed yield/plant (0.348–0.389), obtained low (< 0.400) prediction accuracy (Tables 1, 4). In the current study, all the traits showed high heritability (> 75%) except protein content (57.4%). It is noted that despite achieving high heritability (> 75%) for pods/plant, shelling  %, total yield/plant, yield/ha, protein content, rust@75 days, late leaf spot@75 days, late leaf spot@105 days, pod yield/plant and seed yield/plant, these traits have achieved low prediction accuracy (Table 4).

#### Performance of different GS models for untested genotypes environment (CV1 scheme)

In CV1, the model E + L yielded negative prediction values for all the traits studied. Among other three GS models, the prediction values across four environments were found less consistent for CV1 scheme (Table 1) as compared to CV0. The exceptions in consistent prediction with all the four models were observed for pods/plant, pod yield/plant, shelling  %, and hundred seed weight for Env1; days to 50% flowering and plant height, rust@90 days and late leaf spot@75 days for Env2 (Jalgaon, Rainy 2015); and pods/plant and palmitic acid for Env3 while days to maturity, plant height, pod yield/plant, hundred seed weight, seed yield/plant, total yield/plant, yield/ha, oil content and protein content for Env4 (Patancheru, Post-Rainy 2015–2016) (Table 2).

The high prediction accuracy (> 0.600) across the three models was observed for only for disease scores, i.e., rust@90 days (0.623–0.624), rust@105 days (0.638–0.646) and late leaf spot@90 days (0.624–0.629) (Tables 2, 4). A majority of the traits, namely days to 50% flowering (0.501–0.503), days to maturity (0.466–0.489), primary branches/plant (0.531–0.540), pods/plant (0.453–0.471), pod yield/plant (0.374–0.423), hundred seed weight (0.430–0.469), seed yield/plant (0.375–0.422), total yield/plant (0.486–0.533), yield/ha (0.486–0.533), oleic acid (0.493–0.496), linoleic acid (0.466–0.468), OLR (0.469–0.473), palmitic acid (0.465–0.468), rust@75 days (0.445–0.488), late leaf spot@75 days (0.465–0.495) and late leaf spot@105 days (0.558–0.579), have obtained medium (0.400–0.600) prediction accuracy. The low (< 0.400) prediction has been observed for plant height (0.367–0.373), shelling  % (0.326–0.335), stearic acid (0.254–0.273), oil content (0.344–0.362) and protein content (0.146–0.222) (Tables 1, 4). Among the high heritable traits (*h* > 75%), only rust@90 days, rust@105 days and late leaf spot@90 days achieved high prediction accuracy (Table 4).

#### Performance of different GS models for unevaluated environment (CV2 scheme)

In general, the prediction values across four environments with four GS models were found consistent for CV2 scheme (Table 3). The exceptions to consistent prediction with all the four models were observed for pod yield/plant, and seed yield/plant in Env1; days to 50% flowering, plant height, hundred seed weight, rust@75 days, rust@90 days, late leaf spot@75 days and late leaf spot@90 days for Env2 (Jalgaon, Rainy 2015); and days to maturity, plant height, shelling  %, hundred seed weight, seed yield/plant, total yield/plant, yield/ha, stearic acid and oil content for Env4 (Patancheru, Post-Rainy 2015–2016) (Table 3).

The high prediction accuracy (> 0.600) across the four models was observed for days to 50% flowering (0.657–0.672), days to maturity (0.731–0.769), primary branches/plant (0.675–0.695), plant height (0.640–0.659), shelling  % (0.468–0.505), hundred seed weight (0.670–0.721), oleic acid (0.787–0.791), linoleic acid (0.762–0.769), OLR (0.757–0.765), palmitic acid (0.820–0.826), stearic acid (0.717–0.738), oil content (0.672–0.699), rust@90 days (0.744–0.756), rust@105 days (0.718–0.751) and late leaf spot@90 days (0.710–0.735) (Tables 1, 4). The traits, namely pods/plant (0.434–0.511), total yield/plant (0.499–0.603), yield/ha (0.499–0.603), protein content (0.411–0.461), rust@75 days (0.489–0.541), late leaf spot@75 days (0.499–0.538) and late leaf spot@105 days (0.572–0.654), have obtained medium (0.400–0.600) prediction accuracy. The low (< 0.400) prediction has been observed for pod yield/plant (0.321–0.454) and seed yield/plant (0.336–0.462) (Tables 3, 4). Among the high heritable traits (> 75%), pod yield/plant and seed yield/plant showed low prediction accuracy (Table 4).

### Comparative prediction accuracy across models and cross-validation schemes

Among four GS models tested for 24 traits, the model (E + L) (0.613) performed marginally better in general for all the traits as compared to models (E + L + G) (0.571), (E + L + G + GE) (0.577) and (E + L + G + LE + GE) (0.581) (Table 5). The model (E + L) completely failed in cross-validation scheme CV1, and it yielded negative predictions. In general, the predictions were consistent across different models and cross-validation schemes (except model M1 for CV1) for different traits. However, there have been large variations in predictions obtained for different traits. For example, the palmitic acid (0.704), rust@90 days (0.705), rust@105 days (0.708) followed by days to 50% flowering (0.614), days to maturity (0.653), primary branches/plant (0.639), hundred seed weight (0.613), oleic acid (0.692), linoleic acid (0.668), OLR (0.666), late leaf spot@90 days (0.694) and late leaf spot@105 days (0.602) showed high (> 0.600) genomic prediction (Table 5). The traits, namely pod yield/plant (0.402), seed yield/plant (0.408) and protein content (0.354), showed lowest predictions among the studies traits. The remaining traits showed medium prediction accuracies. The results also indicated absence of relationship between trait heritability and its prediction accuracy.

**Table 5:** Comparative prediction accuracy for different traits by four models under three cross-validation schemes in groundnut

Traits	Cross-validation schemes				GS models				
	CV0	CV1	CV2	Mean	E + L	E + L + G	E + L + G + GE	E + L + G + GE + LE	Mean
Days to 50% flowering (FLOW50)	0.666	0.502	0.668	0.612	0.658	0.616	0.611	0.613	0.614
Days to maturity (DM)	0.724	0.481	0.750	0.652	0.731	0.635	0.663	0.660	0.653
Primary branches/plant (NPB)	0.686	0.536	0.688	0.637	0.677	0.637	0.641	0.640	0.639
Pods/plant (NPP)	0.469	0.465	0.486	0.473	0.438	0.475	0.484	0.487	0.482
Plant height (PH)	0.646	0.371	0.652	0.556	0.643	0.556	0.558	0.560	0.558
Pod yield/plant (PYPP)	0.357	0.407	0.405	0.390	0.328	0.383	0.405	0.416	0.402
Shelling % (SHP)	0.483	0.332	0.492	0.436	0.471	0.435	0.441	0.441	0.439
Hundred seed weight (HSW)	0.675	0.456	0.698	0.610	0.672	0.595	0.622	0.623	0.613
Seed yield/plant (SYPP)	0.370	0.406	0.416	0.397	0.342	0.389	0.413	0.423	0.408
Total yield/plant (TYPLT)	0.520	0.517	0.562	0.533	0.503	0.521	0.547	0.556	0.542
Yield/ha (YPH)	0.520	0.517	0.562	0.533	0.503	0.521	0.547	0.556	0.542
Oleic acid (OA)	0.790	0.495	0.790	0.692	0.787	0.692	0.693	0.693	0.692
Linoleic acid (LA)	0.767	0.468	0.767	0.667	0.763	0.666	0.668	0.669	0.668
Oleic/linoleic acid ratio (OLR)	0.762	0.471	0.762	0.665	0.758	0.665	0.666	0.666	0.666
Palmitic acid (PA)	0.822	0.466	0.823	0.704	0.821	0.703	0.705	0.705	0.704
Stearic acid (SA)	0.719	0.266	0.728	0.571	0.718	0.564	0.576	0.577	0.572
Oil content (OC)	0.675	0.356	0.686	0.572	0.673	0.566	0.577	0.579	0.574
Protein content (PC)	0.419	0.196	0.438	0.351	0.415	0.327	0.367	0.367	0.354
Rust@75 days (RUST75)	0.502	0.457	0.508	0.489	0.516	0.518	0.464	0.477	0.487
Rust@90 days (RUST90)	0.743	0.624	0.749	0.705	0.745	0.711	0.700	0.705	0.705
Rust@105 days (RUST105)	0.730	0.643	0.740	0.704	0.719	0.707	0.707	0.710	0.708
Late leaf spot@75 days (LLS75)	0.534	0.477	0.525	0.512	0.532	0.538	0.491	0.505	0.511
Late leaf spot@90 days (LLS90)	0.719	0.627	0.728	0.691	0.712	0.695	0.689	0.697	0.694
Late leaf spot@105 days (LLS105)	0.589	0.571	0.625	0.595	0.576	0.597	0.601	0.608	0.602
Average	0.620	0.463	0.635	0.573	0.613	0.571	0.577	0.581	0.576

## Discussion

Breeding methodologies have been evolving over the time to develop superior crop varieties for achieving higher productivity to feed the global population. Majority of the breeding programs have been relying on phenotype-based selection approaches with some efforts dedicated toward using marker-assisted selection (MAS) or marker-assisted backcrossing (MABC) including groundnut (Pandey et al. 2016; Varshney 2016; Varshney et al. 2019). The MAS and MABC efforts are now routine in few groundnut breeding programs; however, these breeding methods are mostly successful for simple traits for which diagnostic markers are being developed through trait mapping approaches (Pandey et al. 2020). The major problem lies with complex traits for which generating precise and repeatable phenotyping data for complex traits is challenging as a consequence of high G × E interaction. Under such scenario, a new breeding approach called genomic selection is gaining momentum across crops which promises to improve complex as well as simultaneous improvement of multiple traits (Meuwissen et al. 2001; Jannink et al. 2010; Crossa et al. 2017). This approach uses genome-wide marker and multi-environment phenotyping data on target complex traits on a training population possessing diversity for target traits and close resemblance with the candidates under selection.

The availability of cost-effective high- to mid-density genotyping assays is very important for deploying genomic selection in any crop species. The groundnut, one of the most important food and oilseed crops of the world, has recently attained optimum genomic resources such as the reference genomes for diploid progenitors (Bertioli et al. 2016; Chen et al. 2016) and both the subspecies of cultivated tetraploid (Bertioli et al. 2019; Chen et al. 2019; Zhuang et al. 2019) in addition to a high-density genotyping assay (Axiom_Arachis array with 58 K SNPs) (Pandey et al. 2017; Clevenger et al. 2017). These optimum genomic resources have accelerated the process and precision in several genomics and breeding applications including initiating genomic selection in groundnut. In this context, a training population in groundnut was constituted successfully with 340 elite lines containing several desired agronomic features required for Indian and other global breeding programs. The results clearly showed high variability for traits targeted in this effort, and the high-density genotyping assay played important role in performing genomic prediction for these target traits. Therefore, this panel has potential to serve as ideal training population for different Indian groundnut breeding programs.

Conventional breeding relies on phenotype-based selections for complex traits performing replicated yield trials in advanced (F6 onward) generations which require huge resources to grow large number of plants in each generation and conduct replicated yield trials. GS provides an advantage by facilitating selection of promising individuals at very early generations (F2), thereby reducing the number of lines to be generation advanced and phenotyped in replicated yield trials. If rapid generation advancement technology is integrated with this approach, GS also will save time by shortening breeding cycle in addition to offering more precise selection and reduced use of resources in the breeding process (Heffner et al. 2009, 2011; Isidro et al. 2015). There have been several studies on this approach which clearly indicated that GS is affected by several factors such as marker types and density (Chen and Sullivan 2003; Poland and Rife 2012; Zhang et al. 2017; Norman et al. 2018; Roorkiwal et al. 2018), population size (Daetwyler et al. 2010; Zhang et al. 2017; Norman et al. 2018), marker types and statistical models (Heslot et al. 2012; Roorkiwal et al. 2018). Besides above important considerations, the main question which has been lingering on was that GS breeding can be made more effective to tackle G × E interactions while performing genomic-based predictions for complex traits. In this context, this study reports constituting a training population in groundnut, genotyping with high-density SNP array and testing four GS models under three different cross-validation schemes in groundnut. This study provides information on prediction accuracy for four important GS models which can take care of G × E interactions for performing more precise selection in GS breeding in groundnut. The identified best prediction models from this study are now ready for deployment in routine GS breeding as the impact of G × E interactions in the precision of selecting best performing plants has been accounted for the models.

It is very difficult for any breeding program to generate phenotyping data on training population at all the possible evaluation sites. Under such circumstances, the crop breeder may face multiple situations on their datasets for training population such as (a) lines have never been evaluated/phenotyped in any of the target environment, (b) lines of the training population may have been phenotyped in some environments but not all the environments, and (c) no phenotyping data have been generated for some environments. To address the situation (a), we used a cross-validation scheme (CV1) to assess the prediction accuracy for the situation where a set of lines have never been evaluated/phenotyped in any of the target environment to see whether these GS models can give high prediction accuracy for the unevaluated genotypes in different environments by taking clues from only genotyping data. The results from this study clearly showed total reliance on genomic information for achieving high prediction accuracy under such situation, and one of the models (M1) fell flat with very poor prediction accuracy as it does not use genomic information, while model 2 (M2) may not be good to use for achieving higher prediction for the location with high G × E. The results showed that remaining two GS models were competitive in achieving high prediction accuracies, indicating their potential deployment in GS breeding under such situations with high G × E.

To address situation (b), the cross-validation scheme CV2 was used to assess the prediction accuracy for the situation where some lines of a larger set have been evaluated in only few environments (i.e., not in all the target environments). The idea was to see performance of these GS models to assess prediction accuracy for untested lines and unobserved environments using the information from evaluated lines in different environments. The results from current study clearly showed comparative performance of all the four candidate GS models which indicated that such scenario can be handled with ease using any of these prediction models. It also indicates that breeder can introduce new germplasm with partial datasets into the extended training population and there would not be any adverse impact on prediction accuracies, and thus, selection efficiency will not be affected. Although the models showed good prediction accuracies in predicting the performance of genotypes in untested environments, it will not completely eliminate the need of testing especially in advanced generations; therefore, the real-time testing of promising lines would be needed prior to product advancement. However, in such scenario GS would be useful in reducing the resources for real-time testing of low performing genotypes in respective target environments and facilitate to identify the best suitable genotypes for testing in different target production environments. Similarly, to address the situation (c), the cross-validation scheme CV0 was used to assess the prediction accuracy for unobserved environment using the phenotyping information on training set from related or remaining environments. In this case, prediction was made for each environment using the information from remaining environments. Similar to CV2 scenario, the results from current study for CV0 also demonstrated comparative performance of all the four candidate GS models which indicated that breeder can introduce new environment into the ongoing breeding program without any adverse impact on prediction accuracies and selection efficiency. Similar results have also been obtained in other studies in different crops (de los Campos et al. 2009; Hays and Goddard 2010; Heffner et al. 2009; Gorjanc et al. 2016) including chickpea (Roorkiwal et al. 2018) for these three scenarios, and the results obtained in this study, therefore, provide more confidence while deploying this scheme in case of groundnut.

Among the agronomic traits, days to maturity, pods/plant, shelling  %, hundred seed weight and yield/ha along with nutritional quality traits such as oil content and protein content are the key priority traits in groundnut governed by polygenes and are complex in nature. However, the resistance to LLS and rust in groundnut are governed by major quantitative trait loci (Sujay et al. 2012; Kolekar et al. 2016; Shirasawa et al. 2018) and used for introgression of LLS and rust resistance into elite varieties (Varshney et al. 2014; Janila et al. 2016; Shasidhar et al. 2020). The quantitative inheritance with additive effect of minor genes has been reported for LLS and rust resistance in groundnut (Janila et al. 2013). Furthermore, the high G × E interactions and environment effect make these traits more complex in nature. Hence, for achieving higher genetic gains for resistance to LLS and rust, both major and minor QTL/gene effects need to be captured that can be very well taken care in GS. The models considering G × E interactions in prediction of GEBVs would be of great use to develop product with wider adaptability.

Identification of best performing GS prediction model is the critical question to be answered before initiating GS breeding. The current study tested four GS models, i.e., E + L, E + L + G, E + L + G + GE (naïve interaction model), and E + L + G + LE + GE (naïve and informed interaction model) (de los Campos et al. 2013; Pérez-Rodríguez et al. 2015). The results showed that high prediction accuracies can be achieved for CV0 and CV2 scenarios with best performance from the naïve and informed interaction model performed followed by informed interaction model and main-effect model E + L + G. One of the main-effect models (E + L) which does not use genotyping information has completely failed in prediction for cross-validation scheme (CV1) to assess the prediction accuracy, while the remaining three GS models, although performed much better than model E + L, performed poorly in providing good prediction for untested genotypes. Therefore, achieving high prediction accuracy for this scenario is still a distant dream and more suitable models need to be developed and tested to predict the performance of genotypes in untested environments. Besides selection of parents, the prediction of GEBVs of newly developed lines which are not tested in any environment is one of the major applications of GS in the breeding programs. The low prediction accuracies for CV1 could be attributed to low resemblance between the training set and candidate population. The prediction accuracies can be substantially increased by adding more lines in training population that shows genetic resemblance with candidate population. These models have shown very good performance for simple and complex traits tested in this research and therefore can also be extended to other complex traits in groundnut such as heat tolerance and aflatoxin contamination (Pandey et al. 2019). It is worth mentioning that the models which consider G × E effects hold high potential in improving further the prediction accuracies (Jonas and de Koning 2013; Oakey et al. 2016; Roorkiwal et al. 2018); therefore, such models may be more appropriate to deploy in GS breeding.

In summary, this study reports the development and testing of four GS models and provides comparative performance under three important cross-validation which occur more frequently before breeders due to several reasons such as lack of resources, time, facility or inclusion of new potential parents/traits/environments in breeding program. The current study tested four GS models, i.e., E + L, E + L + G, E + L + G + GE (naïve interaction model), and E + L + G + LE + GE (naïve and informed interaction model), and suggests use of latter two models for achieving higher prediction accuracies for even traits with large G × E effects in groundnut. The identified GS models could be deployed in breeding program upon validation of prediction accuracies on candidate population.

## Electronic supplementary material

Below is the link to the electronic supplementary material.

Supplementary Table 1 Details of genotypes of groundnut training population (XLSX 34 kb)

Supplementary Table 2 Details of SNPs genotyped through Axiom_Arachis SNP array (XLS 13,064 kb)

Supplementary Table 3 Genetic parameters of phenotyping traits across locations (XLSX 18 kb)
